# Association between intake of different types of kimchi and dyslipidemia in Korean adults: a prospective cohort study

**DOI:** 10.3389/fnut.2026.1784228

**Published:** 2026-05-25

**Authors:** Seok-Jae Oh, Kwon Woo Nam, Sangah Shin

**Affiliations:** Department of Food and Nutrition, Chung-Ang University, Anseong-si, Gyeonggi-do, Republic of Korea

**Keywords:** cohort study, dyslipidemia, hypercholesterolemia, kimchi, Korean adult

## Abstract

**Background:**

Dyslipidemia poses a major public health risk worldwide. Although numerous studies have suggested that dietary factors are related to dyslipidemia, epidemiological studies examining the relationship between kimchi consumption and dyslipidemia in large populations remain limited. In this study, we aimed to evaluate the association between kimchi consumption and dyslipidemia.

**Methods:**

In total, 4,666 participants were recruited from the Ansan-Anseong cohort. Total kimchi and baechu (Napa cabbage) kimchi consumption was calculated using a validated semi-quantitative food frequency questionnaire. The association between kimchi consumption and dyslipidemia, along with its four key indicators, was evaluated using the Cox proportional hazards analysis.

**Results:**

In women, the risk of hypo-high-density lipoprotein (hypo-HDL) cholesterolemia was reduced in the group that consumed the most baechu kimchi [≥3 servings/day; hazard ratio (HR): 0.778; 95% confidence interval (CI): 0.671–0.981]. Compared with the group that consumed the least amount of baechu kimchi, the group that consumed 1–2 servings/day of baechu kimchi showed a decreased risk of hypercholesterolemia in men (HR: 0.614; 95% CI: 0.398–0.948) and hypo-HDL-cholesterolemia in women (HR: 0.797; 95% CI: 0.649–0.979).

**Conclusion:**

These findings highlight the importance of moderate kimchi intake in the prevention and management of cholesterol level.

## Background

Dyslipidemia is a well-known risk factor for cardiovascular disease (CVD), a major non-communicable cause of death (1, 2). While the age-standardized global mortality rate of CVD has been declining, the number of CVD-related deaths has increased by approximately 41% during the same period (3). Therefore, prevention of dyslipidemia is important for mitigating public health risk. Dyslipidemia is characterized by high levels of triglycerides (TG), total cholesterols (TC), and low-density lipoprotein cholesterol (LDL-C) and low levels of high-density lipoprotein cholesterol (HDL-C) in the blood (4). When examining the trend of age-standardized prevalence rates among Korean adults between 2007 and 2020, hypercholesterolemia rates increased from 8.8 to 19.9%, and hyper-LDL-cholesterolemia rates increased from 8.7% to 19.1% (4).

Improving diet is a key strategy for reducing risk factors associated with dyslipidemia (5). Kimchi is a traditional Korean fermented food made from baechu (cabbage), radish, and other vegetables and seasonings such as chives, ground garlic, red pepper powder, ground ginger, leek, green onion, salt, and fermented seafood (*Jeotgal* and *Aekjot*) (6, 7). Kimchi is produced through lactic acid bacteria (LAB) fermentation of its ingredients, involving representative strains such as *Lactobacillus*, *Weissella*, and *Leuconostoc* (8, 9). Owing to the probiotic effect of LAB produced during kimchi fermentation, it exhibits anti-cancer, anti-obesity, and anti-atherosclerotic properties (8). In a clinical study involving 90 overweight participants with body mass index (BMI) between 23 and 30 kg/m^2^ who consumed 3,000 mg daily of kimchi powder for 12 weeks, the group consuming kimchi fermented with *Leuconostoc mesenteroides* KCKM0828 (LMS-K) showed a significant decrease in LDL-C levels, coupled with a notable increase in HDL-C levels (10). Mice fed a high-fat diet (HFD) and orally administered spontaneously fermented kimchi or starter culture-fermented kimchi (LMS-K) exhibited serum TG, TC, and LDL-C levels that were significantly lower in the kimchi group than in the HFD group (11). Kimchi is also low in calories and fats and contains various nutrients such as fiber, vitamins, and minerals (including calcium, iron, phosphorus, and selenium) (12). A previous study showed that kimchi ingredients, such as garlic and onions, and nutrients such as dietary fiber, may contribute to improved serum lipid levels (13). However, these findings have largely been derived from experimental or short-term clinical settings, and whether such lipid-related effects of kimchi are observable at the population level remains unclear.

Despite the beneficial effects of kimchi, epidemiological studies examining its association with dyslipidemia are lacking. In this study, we aimed to evaluate the associations of kimchi consumption with dyslipidemia in Korean adults, determine the optimal consumption level, and investigate the relationship between total kimchi intake across various types of kimchi and dyslipidemia.

## Methods

### Study population

This cohort study was based on the Ansan-Anseong cohort study, a longitudinal survey targeting the local population that aims to examine the association between genetic, lifestyle and environmental factors and the frequency of metabolic and chronic diseases in South Koreans. It is funded by the South Korean government (Korean National Research Institute of Health, Korean Centers for Disease Control and Prevention, and the Ministry of Health and Welfare). Information regarding the study procedures and protocols can be found in a previous publication (14). The baseline survey was conducted between June 2001 and January 2003, involving 10,030 Koreans aged ≥40 years living in Ansan and Anseong. Health assessments for demographic, anthropometric, social, physical, and medical information were conducted at a tertiary hospital in Ansan City. Nine follow-up evaluations of participants who completed the baseline survey were conducted biennially through visits until 2020, and written consent was obtained from all participants during each visit. This study was approved by the Ethics Committee of the Korean Health and Genomic Study of the Korean National Institutes of Health, and the institutional review boards of all participating hospitals (IRB No. 1041078-20230628-HR-174).

**Figure 1:**
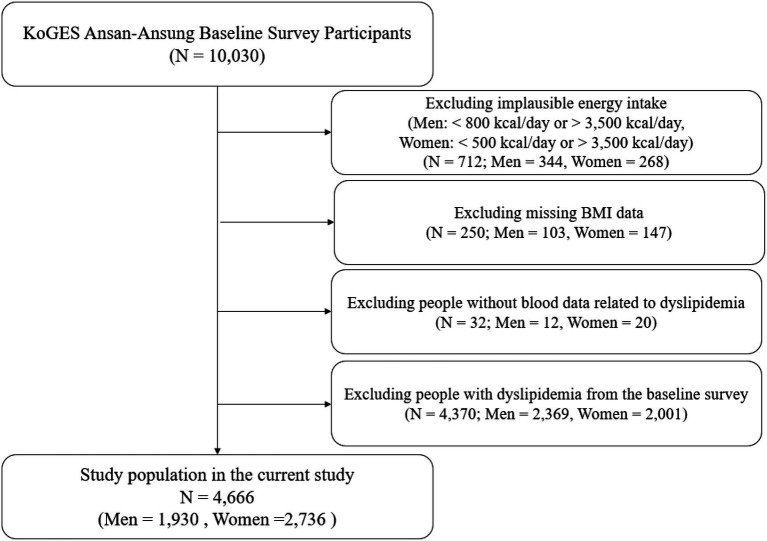
Flow chart of the study population. BMI, body mass index.

In the present study, participants who completed at least one follow-up survey after the baseline survey were included. Furthermore, we excluded participants with implausible energy intake (<800 or >3,500 kcal per day for men: 344, <500 or >3,500 kcal per day for women: 268) (*n* = 712) (15), missing BMI data (men: 103 and women: 147) (*n* = 250), no blood data related to dyslipidemia (men: 12 and women: 20), and a diagnosis of dyslipidemia at baseline survey (men: 2,369 and women: 2,001) (*n* = 4,370). Consequently, a total of 4,666 participants (men: 1,930 and women: 2,736) were included in the final analysis. The detailed flowchart of this process is shown in Figure 1.

### Dietary assessment

Dietary intake was assessed using a validated and reproducible 103-item semi-quantitative food frequency questionnaire (FFQ) developed by the KoGES and Ansan-Anseong cohort study (16). In the FFQ, kimchi was classified into four categories: baechu kimchi, kkakdugi, nabak kimchi/dongchimi, and other kimchi types. In this study, we analyzed baechu kimchi, which is widely consumed, and total kimchi, which comprise four types of kimchi.

Kimchi intake was calculated in grams per day using the FFQ from the responses to frequencies and quantities. The nine frequency categories were as follows: never or seldom, once a month, 2–3 times a month, 1–2 times a week, 3–4 times a week, 5–6 times a week, once a day, twice a day, and 3 times a day. The serving sizes of kimchi intake were categorized as 0.5, 1, or 2 servings. We assessed kimchi intake based on the serving size per day. According to the Manual of the Korean Genome and Epidemiology Study and a validated semi-quantitative FFQ (SQFFQ) (16, 17). The serving size was set at 50 g for baechu kimchi, kkakdugi, and other types of kimchi, whereas nabak kimchi and dongchimi were assigned a serving size of 95 g considering their higher water content. Total kimchi intake was calculated as the sum of the four types of kimchi consumed. Total kimchi intake was categorized as less than 1 serving/day, 1–2 servings/day, 2–3 servings/day, 3–5 servings/day, and 5 or more servings/day. Baechu kimchi intake was categorized as less than 1 serving/day, 1–2 servings/day, 2–3 servings/day, and 3 or more servings/day.

### Definition of dyslipidemia

The outcome of interest in the present study was the occurrence of dyslipidemia and its associated elements, including hypercholesterolemia, hyper-LDL-cholesterolemia, hypertriglyceridemia, and hypo-HDL-cholesterolemia, during the period from the baseline survey to follow-up survey. All blood samples were collected after 8 h of fasting, and dyslipidemia along with its four components was diagnosed (14).

Participants who met one or more of the following four criteria were diagnosed with dyslipidemia. Hypercholesterolemia was defined as a blood total cholesterol level ≥240 mg/dL, hyper-LDL-cholesterolemia was defined as a blood LDL-C level ≥160 mg/dL, and hypertriglyceridemia was defined as a TG level ≥200 mg/dL. Conversely, hypo-HDL-cholesterolemia was defined as a blood HDL-C level of <40 mg/dL. The criteria for these four components can be found in a previous publication (4).

### Covariates

Covariates including sociodemographic variables, including age, BMI, income level, education level, and marital status and lifestyle variables such as smoking status, alcohol consumption, and physical activity were collected in the Ansan-Anseong cohort study using a self-administered questionnaire. BMI was calculated as weight divided by height squared (kg/m^2^). Household income was divided into two groups (<3 million won and ≥3 million won per month). Educational level was classified into three categories: less than middle school, high school, and college or above. Marital status was divided into married and others. Current smoking status was classified into three categories: non-smokers, past smokers (cigarettes smoked in the past but not smoking at the time of the survey), and current smokers (18). Alcohol consumption was divided into non-drinkers (never or abstained for >2 months) and current drinkers Physical activity information was collected using the International Physical Activity Questionnaire (IPAQ) form, and metabolic equivalent (MET) was assigned according to the Adult Compendium of Physical Activities (19). The high physical activity group included individuals who engaged in vigorous-intensity activity on ≥3 days per week accumulating ≥1,500 MET-min/week, or those who performed ≥7 days of any combination of walking, moderate- or vigorous-intensity activities achieving ≥3,000 MET-min/week. The moderate physical activity group comprised individuals who met any of the following criteria: (1) vigorous-intensity activity for ≥20 min per day on ≥3 days per week; (2) moderate-intensity activity and/or walking for ≥30 min per day on ≥5 days per week; or (3) ≥ 5 days of any combination of walking, moderate-, or vigorous-intensity activities achieving ≥600 MET-min/week. Individuals who did not meet any of the criteria for the moderate or high groups were classified into the low physical activity group (20). Finally, participants were categorized into three groups based on their physical activity levels: low, moderate, and high, as defined by the corresponding MET-min/week and activity duration (21).

### Statistical analysis

All statistical analyses were stratified by sex to determine the association between kimchi intake and dyslipidemia. Participants were categorized into four groups according to their baechu kimchi intake. To evaluate the differences in general characteristics according to baechu kimchi intake, we used the chi-squared test for categorical variables (percentages) and linear regression for continuous variables (mean ± standard deviation). We performed Cox proportional hazards regression analysis to estimate the hazard ratios (HR) and 95% confidence intervals (CI) of dyslipidemia and its four components according to baechu kimchi intake after adjusting for potential confounders. The first model was adjusted only for age (continuous). Multivariable-adjusted model 1 was adjusted for BMI (continuous), income level, education level, marital status, smoking status, alcohol consumption, physical activity, and energy intake (continuous). Multivariable-adjusted model 2 was adjusted for energy-adjusted sodium intake (continuous) and the sum of rice intake (continuous, cooked white rice, cooked white rice with multiple-grains, and cooked white rice with barley). All statistical analyses were performed using SAS (version 9.4; SAS Institute, Cary, NC, USA) and a two-sided *p*-value <0.05 was considered statistically significant.

## Results

In total, 4,666 participants (1,930 men and 2,736 women) were included in this study. Table 1 shows the baseline general characteristics according to baechu kimchi intake stratified by sex. Of the study participants, 52.59% of men and 42.36% of women consumed the most baechu kimchi (≥3 servings/day). Among men, the group that consumed the most baechu kimchi included significantly more current drinkers than the group that consumed less than one serving of baechu kimchi per day (*p* = 0.0079).

**Table 1:** General characteristics of the study population at baseline survey according to baechu kimchi consumption and stratified by sex.

	Baechu kimchi consumption									
	Men (*n* = 1,930)					Women (*n* = 2,736)				
	<1 serving/day (*n* = 222)	1–2 servings/day (*n* = 571)	2–3 servings/day (*n* = 122)	≥3 servings/day (*n* = 1,015)	*p* value	<1 serving/day (*n* = 401)	1–2 servings/day (*n* = 951)	2–3 servings/day (*n* = 225)	≥3 servings/day (*n* = 1,159)	*p* value
Age (years)	53.35 ± 9.16	54.56 ± 9.24	48.87 ± 8.08	51.24 ± 8.79	<0.0001	50.75 ± 9.13	52.77 ± 8.98	48.03 ± 7.73	50.74 ± 8.54	<0.0001
Age										
40–49 years	90 (40.54)	209 (36.60)	80 (65.57)	538 (53.00)	<0.0001	227 (56.61)	417 (43.85)	152 (67.56)	635 (54.79)	<0.0001
50–59 years	64 (28.83)	149 (26.09)	21 (17.21)	245 (24.14)		76 (18.95)	271 (28.50)	47 (20.89)	269 (23.21)	
60–69 years	68 (30.63)	213 (37.30)	21 (17.21)	232 (22.86)		98 (24.44)	263 (27.66)	26 (11.56)	255 (22.00)	
Obesity										
BMI^1^(kg/m^2^)	23.1 ± 2.92	22.86 ± 2.84	23.33 ± 2.65	23.57 ± 2.8	<0.0001	24.08 ± 3.12	24.29 ± 3.26	24.56 ± 3.31	24.43 ± 3.22	0.1925
Underweight	14 (6.31)	33 (5.78)	3 (2.46)	29 (2.86)	0.0029	6 (1.50)	21 (2.21)	4 (1.78)	17 (1.47)	0.2689
Normal	98 (44.14)	269 (47.11)	52 (42.62)	411 (40.49)		156 (38.90)	334 (35.12)	68 (30.22)	372 (32.10)	
Overweight	55 (24.77)	129 (22.59)	38 (31.15)	265 (26.11)		99 (24.69)	232 (24.40)	64 (28.44)	315 (27.18)	
Obese	55 (24.77)	140 (24.52)	29 (23.77)	310 (30.54)		140 (34.91)	364 (38.28)	89 (39.56)	455 (39.26)	
Income level										
<3 million won	185 (84.47)	496 (87.48)	94 (77.69)	780 (77.3)	<0.0001	344 (88.03)	812 (87.31)	175 (79.55)	952 (83.66)	0.0046
≥3 million won	34 (15.53)	71 (12.52)	27 (22.31)	229 (22.7)		48 (11.97)	118 (12.69)	45 (20.45)	186 (16.34)	
Education level										
Under middle school	99 (45.00)	300 (52.72)	45 (36.89)	432 (42.69)	0.0001	251 (63.54)	648 (68.57)	114 (50.89)	747 (64.62)	0.0001
High school	67 (30.45)	180 (31.63)	40 (32.79)	356 (35.18)		113 (28.61)	236 (24.97)	87 (38.84)	340 (29.41)	
Over college	54 (24.55)	89 (15.64)	37 (30.33)	224 (22.13)		31 (7.85)	61 (6.46)	23 (10.27)	69 (5.97)	
Marital status										
Married	209 (94.14)	539 (94.89)	116 (95.08)	974 (96.44)	0.3125	332 (83.21)	819 (86.48)	199 (88.44)	994 (86.14)	0.2719
Others	13 (5.86)	29 (5.11)	6 (4.92)	36 (3.56)		67 (16.79)	128 (13.52)	26 (11.56)	160 (13.86)	
Alcohol consumption										
Non-drinker	61 (27.48)	157 (27.59)	25 (20.49)	211 (20.83)	0.0079	280 (70.00)	686 (72.29)	143 (63.56)	807 (70.30)	0.0816
Current drinker	161 (72.52)	412 (72.41)	97 (79.51)	802 (79.17)		120 (30.00)	263 (27.71)	82 (36.44)	341 (29.70)	
Current smoking status										
Never smoker	39 (17.65)	120 (21.05)	25 (20.66)	212 (20.97)	0.1291	367 (94.83)	907 (96.08)	214 (95.96)	1,093 (95.79)	0.9582
Past smoker	69 (31.22)	154 (27.02)	35 (28.93)	339 (33.53)		6 (1.55)	11 (1.17)	2 (0.90)	12 (1.05)	
Current smoker	113 (51.13)	296 (51.93)	61 (50.41)	460 (45.50)		14 (3.62)	26 (2.75)	7 (3.14)	36 (3.16)	
Physical activity										
Low	61 (27.85)	139 (24.60)	37 (30.33)	292 (29.08)	0.0555	134 (34.27)	282 (30.13)	80 (35.71)	391 (34.09)	0.0086
Moderate	41 (18.72)	137 (24.25)	28 (22.95)	264 (26.29)		107 (27.37)	275 (29.38)	83 (37.05)	330 (28.77)	
High	117 (53.42)	289 (51.15)	57 (46.72)	448 (44.62)		150 (38.36)	379 (40.49)	61 (27.23)	426 (37.14)	

One serving size of baechu kimchi was defined as 50 g.

Values are mean ± SD or *n* (%); *p*-values were calculated using the chi-square test for categorical variables and general linear regression for continuous variables.

Values are presented excluding missing data. Missing values were as follows: Income level (men, *n* = 14; women, *n* = 56), Education level (men, *n* = 7; women, *n* = 16), Marital status (men, *n* = 8; women, *n* = 11), Alcohol consumption (men, *n* = 4; women, *n* = 14), Current smoking status (men, *n* = 7; women, *n* = 41), and Physical activity (men, *n* = 20; women, *n* = 38).

1 BMI, body mass index: underweight (<18.5 kg/m^2^), normal (18.5 kg/m^2^ ≤ BMI <23 kg/m^2^), overweight (23 kg/m^2^ ≤ BMI <25 kg/m^2^), and obese (≥25 kg/m^2^).

Table 2 shows the intake of kimchi, macronutrients, and sodium according to the participants’ intake of baechu kimchi. In both men and women, total energy, carbohydrate, protein, fat, and sodium intake tended to increase along with increased baechu kimchi intake (all *p* < 0.0001). Additionally, the intake of baechu kimchi, kkakdugi, nabak kimchi/dongchimi, and other kimchi also show increasing trends (all *p* < 0.05).

**Table 2:** Daily kimchi and nutrient intake according to baechu kimchi consumption.

	Baechu kimchi consumption				
	<1 serving/day	1–2 servings/day	2–3 servings/day	≥3 servings/day	*p* value
Men (**n** = 1,930)					
Participants, *n*	222	571	122	1,015	
Median (range), serving/day	0.4 (0.00–0.79)	1.5 (1.00–1.50)	2 (2.00–2.00)	3 (3.00–4.50)	
Food intake (g/day)					
Baechu kimchi	19.3 ± 12.7	69.2 ± 10.5	100.0 ± 0.0	159.6 ± 25.1	<0.0001
Kkakdugi	14.8 ± 20.0	36.3 ± 36.8	40.5 ± 40.0	61.4 ± 64.1	<0.0001
Nabak kimchi/Dongchimi	20.4 ± 33.1	42.0 ± 63.2	36.8 ± 60.1	50.7 ± 85.5	<0.0001
Other kimchi	7.7 ± 16.1	9.6 ± 19.9	10.1 ± 21.4	13.0 ± 28.5	0.0059
Nutrient intake					
Total energy (kcal/day)	1808.3 ± 507.2	1904.7 ± 502.3	1908.2 ± 528	2009.3 ± 462.8	<0.0001
Carbohydrates (g/day)	312.0 ± 82.1	334.9 ± 84.6	323.1 ± 89	348.8 ± 74.6	<0.0001
Protein (g/day)	60.3 ± 23.6	63.5 ± 22.4	66.0 ± 21.9	69.6 ± 21.7	<0.0001
Fat (g/day)	32.3 ± 18.3	31.7 ± 16.2	37.0 ± 16.9	35.1 ± 16.3	<0.0001
Sodium^1^ (mg/day)	1688.8 ± 916.1	2659.2 ± 1086.3	3087.0 ± 1116.2	4054.0 ± 1396.3	<0.0001
% Energy from					
Carbohydrates (% of Energy)	70.9 ± 8.7	71.9 ± 6.7	68.8 ± 6.7	70.8 ± 6.5	<0.0001
Protein (% of Energy)	13.3 ± 2.7	13.4 ± 2.3	13.9 ± 2.2	13.8 ± 2.1	<0.0001
Fat (% of Energy)	15.7 ± 6.4	14.7 ± 4.9	17.2 ± 5	15.3 ± 4.8	<0.0001
Women (**n** = 2,736)					
Participants, *n*	401	951	225	1,159	
Median (range), serving/day	0.4 (0.00–0.79)	1.5 (1.00–1.50)	2 (2.00–2.00)	3 (3.00–4.50)	
Food intake (g/day)					
Baechu kimchi	18.8 ± 12.2	69.0 ± 10.6	100.0 ± 0.0	157.2 ± 22.1	<0.0001
Kkakdugi	11.9 ± 20.8	26.0 ± 32.6	34.6 ± 37.3	48.6 ± 60.5	<0.0001
Nabak kimchi/Dongchimi	17.8 ± 42.5	39.0 ± 65.3	25.4 ± 49.7	45.9 ± 83.3	<0.0001
Other kimchi	5.2 ± 16	6.1 ± 17.6	6.7 ± 14.7	10.6 ± 28.8	<0.0001
Nutrient intake					
Total energy (kcal/day)	1649.1 ± 565.5	1801.4 ± 518.3	1697.5 ± 475.2	1922.3 ± 525.9	<0.0001
Carbohydrates (g/day)	292.4 ± 102.5	324.7 ± 92.2	295.3 ± 83.6	343.1 ± 91.3	<0.0001
Protein (g/day)	54.4 ± 23.1	59.4 ± 21.4	58.7 ± 19.0	66.1 ± 22.9	<0.0001
Fat (g/day)	27.0 ± 16.7	27.3 ± 15.6	29.7 ± 14.2	30.2 ± 15.8	<0.0001
Sodium (mg/day)	1560.7 ± 899.3	2452.8 ± 1045.4	2775.8 ± 839.5	3,809 ± 1389.2	<0.0001
% Energy from					
Carbohydrates (% of Energy)	72.2 ± 8.7	73.4 ± 7.0	70.5 ± 6.8	72.5 ± 6.6	<0.0001
Protein (% of Energy)	13.3 ± 2.7	13.2 ± 2.2	13.9 ± 2.1	13.8 ± 2.2	<0.0001
Fat (% of Energy)	14.5 ± 6.4	13.3 ± 5.2	15.5 ± 5.1	13.8 ± 4.9	<0.0001

One serving size of baechu kimchi was defined as 50 g.

Values are mean ± SD or median (min-max); p-value were calculated using the chi-square tests for categorical variables and general linear regression for continuous variables.

1 Sodium was adjusted using the residual method.

Table 3 shows the HR of dyslipidemia and its components according to baechu kimchi intake and sex. After adjustment for confounding covariates, in women, associations with hypo-HDL-cholesterolemia in the 1–2 servings/day (HR: 0.797; 95% CI: 0.649–0.979) and >3 servings/day groups (HR: 0.778; 95% CI: 0.617–0.981), whereas no significant association was observed in the 2–3 servings/day group, and no significant linear trend was identified across intake categories. Compared with the group that consumed the least amount of baechu kimchi, the group that consumed 1–2 servings/day of baechu kimchi showed a decreased risk of hypercholesterolemia in men (HR: 0.614; 95% CI: 0.398–0.948), whereas no significant associations were observed in higher intake groups, and no significant trend was found. Overall, baechu kimchi intake showed some category-specific inverse associations with selected cholesterol-related outcomes, but no consistent dose–response pattern was observed.

**Table 3:** Hazard ratio of dyslipidemia and its components according to baechu kimchi consumption.

	Baechu kimchi consumption (g/day)				
	<1 serving/day	1–2 servings/day	2–3 servings/day	≥3 servings/day	*p* for trend
Men (**n** = 1,930)					
Participants, *n*	222	571	122	1,015	
Median (range), serving/day	0.4 (0.00–0.79)	1.5 (1.00–1.50)	2 (2.00–2.00)	3 (3.00–4.50)	
Person year, mean (sum)	7.4 (1271.8)	7.9 (3440.8)	7.2 (733.5)	8.1 (6156.0)	
Hypertriglyceridemia					
case, *n*	57	145	39	261	
Age-adjusted Model	ref (1.000)	0.957 (0.704–1.300)	1.108 (0.735–1.669)	0.921 (0.690–1.228)	0.5034
Multivariable-adjusted Model 1	ref (1.000)	0.963 (0.706–1.313)	1.163 (0.771–1.755)	0.914 (0.680–1.227)	0.3694
Multivariable-adjusted Model 2	ref (1.000)	0.928 (0.673–1.280)	1.123 (0.730–1.727)	0.842 (0.590–1.202)	0.1462
Hyper-LDL^1^-cholesterolemia					
case, *n*	41	83	28	163	
Age-adjusted Model	ref (1.000)	0.760 (0.523–1.105)	1.142 (0.703–1.854)	0.817 (0.579–1.153)	0.4845
Multivariable-adjusted Model 1	ref (1.000)	0.758 (0.519–1.108)	1.162 (0.715–1.888)	0.823 (0.579–1.17)	0.4494
Multivariable-adjusted Model 2	ref (1.000)	0.746 (0.502–1.109)	1.095 (0.655–1.828)	0.786 (0.508–1.216)	0.5105
Hypercholesterolemia					
case, *n*	38	60	28	123	
Age-adjusted Model	ref (1.000)	0.595 (0.396–0.894)	1.178 (0.720–1.929)	0.645 (0.448–0.931)	0.0906
Multivariable-adjusted Model 1	ref (1.000)	0.593 (0.392–0.895)	1.195 (0.729–1.960)	0.644 (0.442–0.936)	0.0737
Multivariable-adjusted Model 2	ref (1.000)	0.614 (0.398–0.948)	1.234 (0.725–2.100)	0.694 (0.428–1.124)	0.3880
Hypo-HDL^2^-cholesterolemia					
case, *n*	105	262	61	453	
Age-adjusted Model	ref (1.000)	0.917 (0.731–1.150)	1.080 (0.786–1.485)	0.931 (0.752–1.152)	0.6517
Multivariable-adjusted Model 1	ref (1.000)	0.933 (0.741–1.173)	1.120 (0.813–1.543)	0.971 (0.781–1.208)	0.8205
Multivariable-adjusted Model 2	ref (1.000)	0.915 (0.720–1.163)	1.099 (0.786–1.535)	0.933 (0.715–1.217)	0.5543
Dyslipidemia					
case, *n*	139	349	84	605	
Age-adjusted Model	ref (1.000)	0.926 (0.761–1.128)	1.093 (0.832–1.436)	0.924 (0.768–1.112)	0.4693
Multivariable-adjusted Model 1	ref (1.000)	0.937 (0.768–1.144)	1.129 (0.858–1.486)	0.946 (0.783–1.144)	0.5943
Multivariable-adjusted Model 2	ref (1.000)	0.917 (0.745–1.130)	1.103 (0.827–1.469)	0.904 (0.718–1.139)	0.3768
Women (**n** = 2,736)					
Participants, *n*	401	951	225	1,159	
Median (range), serving/day	0.4 (0.00–0.79)	1.5 (1.00–1.50)	2 (2.00–2.00)	3 (3.00–4.50)	
Person year, mean (sum)	8.2 (2542.3)	8.5 (6401.7)	7.8 (1355.9)	8.4 (7584.2)	
Hypertriglyceridemia					
case, *n*	83	192	54	259	
Age-adjusted Model	ref (1.000)	0.870 (0.672–1.126)	1.255 (0.890–1.769)	1.037 (0.810–1.328)	0.3052
Multivariable-adjusted Model 1	ref (1.000)	0.893 (0.687–1.160)	1.245 (0.880–1.762)	1.072 (0.832–1.382)	0.3046
Multivariable-adjusted Model 2	ref (1.000)	0.803 (0.610–1.055)	1.095 (0.763–1.572)	0.866 (0.637–1.176)	0.5892
Hyper-LDL-cholesterolemia					
case, *n*	118	289	84	356	
Age-adjusted Model	ref (1.000)	0.957 (0.772–1.187)	1.332 (1.007–1.763)	1.004 (0.815–1.237)	0.7869
Multivariable-adjusted Model 1	ref (1.000)	0.946 (0.761–1.176)	1.240 (0.934–1.647)	1.003 (0.810–1.242)	0.6696
Multivariable-adjusted Model 2	ref (1.000)	0.962 (0.765–1.210)	1.236 (0.916–1.667)	1.014 (0.778–1.323)	0.4847
Hypercholesterolemia					
case, *n*	110	263	81	331	
Age-adjusted Model	ref (1.000)	0.937 (0.749–1.171)	1.383 (1.037–1.843)	1.005 (0.810–1.247)	0.7016
Multivariable-adjusted Model 1	ref (1.000)	0.936 (0.747–1.173)	1.293 (0.967–1.729)	1.020 (0.817–1.273)	0.5381
Multivariable-adjusted Model 2	ref (1.000)	0.991 (0.780–1.260)	1.368 (1.005–1.864)	1.134 (0.857–1.499)	0.1314
Hypo-HDL-cholesterolemia					
case, *n*	146	352	84	432	
Age-adjusted Model	ref (1.000)	0.889 (0.733–1.079)	1.120 (0.856–1.465)	0.981 (0.813–1.183)	0.6523
Multivariable-adjusted Model 1	ref (1.000)	0.888 (0.730–1.080)	1.080 (0.823–1.417)	0.968 (0.799–1.173)	0.7890
Multivariable-adjusted Model 2	ref (1.000)	0.797 (0.649–0.979)	0.946 (0.714–1.255)	0.778 (0.617–0.981)	0.0969
Dyslipidemia					
case, *n*	242	576	142	722	
Age-adjusted Model	ref (1.000)	0.901 (0.775–1.047)	1.133 (0.920–1.394)	0.995 (0.860–1.151)	0.4699
Multivariable-adjusted Model 1	ref (1.000)	0.894 (0.768–1.041)	1.072 (0.869–1.322)	0.985 (0.848–1.143)	0.5009
Multivariable-adjusted Model 2	ref (1.000)	0.858 (0.731–1.007)	1.003 (0.806–1.249)	0.889 (0.740–1.068)	0.6412

One serving size of baechu kimchi was defined as 50 g.

Multivariable-adjusted Model 1 was adjusted for age, BMI, income level, education level, marital status, smoking status, alcohol consumption, physical activity, and energy intake.

Multivariable-adjusted Model 2 was adjusted for age, BMI, income level, education level, marital status, smoking status, alcohol consumption, physical activity, energy intake, rice intake, and energy-adjusted sodium intake.

The *p* for trend was calculated by assigning median values to each group and was treated as a continuous variable.

1 LDL, low-density lipoprotein.

2 HDL, high-density lipoprotein.

In supplementary analyses, we additionally adjusted the original multivariable model for fruit intake and, separately, for medication and dietary supplement use. Among women, the ≥3 servings/day group showed a significantly reduced risk of hypo-HDL-cholesterolemia both in the model additionally adjusted for fruit intake and in the model additionally adjusted for medication and supplement use. Detailed results are presented in Supplementary Table 2.

## Discussion

We investigated the association between different types of kimchi intake and dyslipidemia in Korean adults and found that for women, consumption of baechu kimchi at 1–2 servings/day or ≥3 servings/day was associated with a reduced risk of hypo-HDL-cholesterolemia, one of the four key indicators of dyslipidemia. In men, consumption of 1–2 servings/day of baechu kimchi was associated with reduced risk of hypercholesterolemia.

Dyslipidemia is a globally recognized and major public health issue (22). Research in Korea conducted in 2022 found that, overall, men have a higher prevalence of dyslipidemia, although postmenopausal women exhibit a prevalence rate similar to that of men (4). Consuming plant-based foods, a traditional global approach, is beneficial for maintaining health, and improving dyslipidemia and other CVD (23). Certain plant-based foods and medicinal plants may also help in managing CVD (24). Modern pharmacological and epidemiological studies have shown that polyphenol consumption lowers blood pressure and improves lipid metabolism, thereby reducing CVD risk (25). These findings suggest that, considering the side effects and costs of drug treatments, dietary management is the safest and most cost-effective approach to prevent diseases with minimal side effects (24).

Kimchi is made by fermenting various vegetables, such as cabbage, radishes or other ingredients, with salt and spices, such as red pepper powder, ground garlic, ground ginger, green onion, and fermented seafood (26). According to research conducted in India, commonly used spices such as garlic, ginger, and onion not only possess antioxidant properties that protect lipids, but the bioactive compounds contained in these spices, such as allicin and capsaicin, have also been shown to improve hypercholesterolemia (27, 28). These results suggest that the spices in kimchi not only enhance flavor but also positively impact CVD risk through antioxidant effects.

Previous studies have shown that LAB produced by fermentation of kimchi positively affects CVD risk (29–31). Among these probiotics, the *L. plantarum EM* strain demonstrated superior cholesterol-lowering effects with higher cell wall concentrations and effectively removed cholesterol in both live and heat-killed states (31). Clinical research has shown that Korean adults who consumed kimchi for 4 weeks had improved blood lipid levels compared with those who did not consume kimchi (30). In this study, we observed that increased kimchi intake improved blood lipid levels in women. Especially, this study conducted an analysis on Korean adults aged 40 and older, thereby including postmenopausal women. It has been reported that menopause in women involves a decrease in the female hormone estrogen, which goes beyond the cessation of menstruation to fundamentally alter the body’s lipid metabolism (32). These findings suggest that kimchi consumption may exert a protective effect against these changes. Additionally, animal experiments have demonstrated that mice consuming juice-containing kimchi’s LAB have reduced cholesterol levels and improved heart health compared to those that did not consume the juice (33). Although this study did not directly assess probiotic activity, prior evidence suggests that LAB produced during kimchi fermentation may contribute to improved lipid metabolism.

Furthermore, the degree of kimchi fermentation has been reported to influence its metabolic effects. A previous study has shown that consumption of fermented kimchi is more effective than fresh kimchi in improving metabolic parameters, including triglyceride (TG) levels, insulin sensitivity, and fasting glucose in humans (26). These effects are thought to be mediated by *Lactobacillus sakei* CJLS03 produced during fermentation, which generates short-chain fatty acids (SCFAs) that migrate to the liver, increase energy expenditure, and suppress lipogenesis (34). In this study, however, no significant association between kimchi intake and TG levels was observed. This may be partly attributable to the inherent variability of TG, which can fluctuate due to short-term dietary intake, lifestyle factors, and emotional states, despite adjustment for sociodemographic variables (35, 36). Additionally, the degree of kimchi fermentation was not distinguished in this study, and these factors should be considered when interpreting the results.

Our findings showed that a trend toward higher kimchi intake increased various nutrients, energy, and sodium. Previous study in the United States of America has indicated that higher salt intake from food is associated with an increased CVD risk (37). Although kimchi is considered a high-sodium fermented vegetable, sodium positively influences its mineral contents and supports the activities of LAB during fermentation (38). In Table 3, which was not adjusted for rice and sodium intake (Model 1), an association with hypercholesterolemia was observed in men. Salt concentration in kimchi is a critical factor in determining the behavior and structure of LAB (39). Among the LAB strains found in kimchi, *Leuconostoc mesenteroides* KDK411 has been shown to lower blood cholesterol levels by absorbing cholesterol and enhancing its excretion through feces (40). Therefore, regardless of the type of salt used in kimchi, it generally has a positive effect on the growth of LAB (38, 39). In addition, recent mechanistic work suggests that cholesterol regulation may involve coordinated hepatic pathways linking lipogenesis, fatty acid oxidation, and bile acid metabolism, including cholesterol disposal (41), which provides broader biologic context for the cholesterol-related associations observed in the present study. For example, Pan et al. reported that hepatic KLF10 protects against hypercholesterolemia through HNF4α-mediated regulation of lipid and bile acid metabolic pathways (42). In Korea, however, kimchi is often consumed alongside other foods or in various prepared dishes, and thus the influence of such consumption contexts on the observed associations cannot be ruled out (37). Therefore, future studies should consider these consumption contexts and may provide further insights into this relationship through more controlled research designs.

This study has some limitations. First, as the study focused on Korean adults ≥40 years, the results may not be generalizable to the entire Korean population. Second, because kimchi is often consumed with various foods, accurately measuring its intake and effects is challenging, and interactions with other foods may alter its health effects. Third, the FFQ used for dietary assessment has limitations, including underreporting or overreporting of total food consumption and selective errors for specific food consumptions.

Despite, this study accurately described the association between kimchi intake and dyslipidemia in South Korea, as it excluded participants with a history of dyslipidemia-related diseases. This study found that moderate kimchi intake, such as 1–2 or ≥ 3 servings/day, was associated with reduced hypercholesterolemia risk in men and hypo-HDL-cholesterolemia risk in women. These results could suggest that moderate kimchi intake could demonstrate the beneficial effects of kimchi spices and LAB in an epidemiological cohort study.

## Conclusion

Baechu kimchi intake was associated with selected lipid parameters according to sex and intake levels; however, no consistent dose–response relationship was observed. These findings may suggest potential associations between kimchi intake and certain lipid-related outcomes but should be interpreted with caution due to the lack of a consistent exposure-response pattern.

## Data Availability

The datasets analyzed for this study are not publicly available due to data privacy and security policies of the Clinical & Omics Data Archive (CODA). However, the data are available from the CODA (https://coda.nih.go.kr/frt/index.do) upon reasonable request and after approval of the institutional review board.
