# Supplementary material for: Association between intake of different types of kimchi and dyslipidemia in Korean adults: a prospective cohort study
Source: Front Nutr. 2026 May 25;13:1784228. doi: 10.3389/fnut.2026.1784228 (PMC13243084; doi:10.3389/fnut.2026.1784228)
Supplement: Supplementary file 1 [file Table_1.DOCX]

**Supplementary Table 1.** Incidence rates of dyslipidemia and its components according to baechu kimchi consumption groups by sex.

|  | Baechu kimchi consumption | | | |
| --- | --- | --- | --- | --- |
|  | <1 serving/day | 1−2 servings/day | 2−3 servings/day | ≥3 servings/day |
| Men (n = 1,930) | 222 | 571 | 122 | 1,015 |
| Person year, mean (sum) | 7.4 (1271.8) | 7.9 (3440.8) | 7.2 (733.5) | 8.1 (6156.0) |
| Hypertriglyceridemia |  |  |  |  |
| Case, n | 57 | 145 | 39 | 261 |
| Incidence rates (per 1,000 person-year) | 44.8 | 42.1 | 53.2 | 42.4 |
| Hyper-LDL^1^-cholesterolemia |  |  |  |  |
| Case, n | 41 | 83 | 28 | 163 |
| Incidence rates (per 1,000 person-year) | 32.2 | 24.1 | 38.2 | 26.5 |
| Hypercholesterolemia |  |  |  |  |
| Case, n | 38 | 60 | 28 | 123 |
| Incidence rates (per 1,000 person-year) | 29.9 | 17.4 | 38.2 | 20.0 |
| Hypo-HDL^2^-cholesterolemia |  |  |  |  |
| Case, n | 105 | 262 | 61 | 453 |
| Incidence rates (per 1,000 person-year) | 82.6 | 13.1 | 83.2 | 73.6 |
| Dyslipidemia |  |  |  |  |
| Case, n | 139 | 349 | 84 | 605 |
| Incidence rates (per 1,000 person-year) | 109.2 | 101.4 | 114.5 | 98.3 |
|  |  |  |  |  |
| Women (n = 2,736) | 401 | 951 | 225 | 1,159 |
| Person year, mean (sum) | 8.2 (2542.3) | 8.5 (6401.7) | 7.8 (1355.9) | 8.4 (7584.2) |
| Hypertriglyceridemia |  |  |  |  |
| Case, n | 83 | 192 | 54 | 259 |
| Incidence rates (per 1,000 person-year) | 32.6 | 30.0 | 39.8 | 34.1 |
| Hyper-LDL^1^-cholesterolemia |  |  |  |  |
| Case, n | 118 | 289 | 84 | 356 |
| Incidence rates (per 1,000 person-year) | 43.3 | 45.1 | 61.9 | 46.9 |
| Hypercholesterolemia |  |  |  |  |
| Case, n | 110 | 263 | 81 | 331 |
| Incidence rates (per 1,000 person-year) | 43.3 | 41.1 | 59.8 | 43.6 |
| Hypo-HDL^2^-cholesterolemia |  |  |  |  |
| Case, n | 146 | 352 | 84 | 432 |
| Incidence rates (per 1,000 person-year) | 57.4 | 55.0 | 62.0 | 57.0 |
| Dyslipidemia |  |  |  |  |
| Case, n | 242 | 576 | 142 | 722 |
| Incidence rates (per 1,000 person-year) | 95.2 | 90.0 | 104.7 | 95.2 |

One serving size of baechu kimchi was defined as 50 g.

^1^LDL, low-density lipoprotein.

^2^HDL, high-density lipoprotein.

**Supplementary Table 2.** Hazard ratio of dyslipidemia and its components according to baechu kimchi consumption

|  | Baechu kimchi consumption (g/day) | | | | |
| --- | --- | --- | --- | --- | --- |
|  | <1 serving/day | 1–2 servings/day | 2–3 servings/day | ≥3 servings/day | P for trend |
| Men (n = 1,930) | 222 | 571 | 122 | 1,015 |  |
| Median (range), serving/day | 0.4 (0.00-0.79) | 1.5 (1.00–1.50) | 2 (2.00–2.00) | 3 (3.00–4.50) |  |
| Person year, mean (sum) | 7.4 (1271.8) | 7.9 (3440.8) | 7.2 (733.5) | 8.1 (6156.0) |  |
| Hypertriglyceridemia |  |  |  |  |  |
| case, n | 57 | 145 | 39 | 261 |  |
| Multivariable-adjusted Model 1 | ref (1.000) | 0.957 (0.704–1.300) | 1.108 (0.735–1.669) | 0.921 (0.690–1.228) | 0.5034 |
| Multivariable-adjusted Model 2 | ref (1.000) | 0.932 (0.674–1.287) | 1.118 (0.726–1.723) | 0.843 (0.590–1.205) | 0.3694 |
| Multivariable-adjusted Model 3 | ref (1.000) | 0.933 (0.677–1.288) | 1.134 (0.737–1.745) | 0.847 (0.593–1.209) | 0.1462 |
| Hyper-LDL^1^-cholesterolemia |  |  |  |  |  |
| case, n | 41 | 83 | 28 | 163 |  |
| Multivariable-adjusted Model 1 | ref (1.000) | 0.751 (0.506–1.117) | 1.104 (0.661–1.844) | 0.796 (0.514–1.232) | 0.4845 |
| Multivariable-adjusted Model 2 | ref (1.000) | 0.762 (0.512–1.136) | 1.118 (0.669–1.869) | 0.810 (0.523–1.256) | 0.4494 |
| Multivariable-adjusted Model 3 | ref (1.000) | 0.765 (0.514–1.139) | 1.124 (0.673–1.879) | 0.817 (0.527–1.266) | 0.5105 |
| Hypercholesterolemia |  |  |  |  |  |
| case, n | 38 | 60 | 28 | 123 |  |
| Multivariable-adjusted Model 1 | ref (1.000) | 0.614 (0.398–0.948) | 1.235 (0.726–2.102) | 0.695 (0.429–1.126) | 0.0906 |
| Multivariable-adjusted Model 2 | ref (1.000) | 0.622 (0.403–0.961) | 1.225 (0.719–2.008) | 0.720 (0.445–1.167) | 0.0737 |
| Multivariable-adjusted Model 3 | ref (1.000) | 0.622 (0.403–0.961) | 1.225 (0.719–2.088) | 0.720 (0.444–1.167) | 0.3880 |
| Hypo-HDL^2^-cholesterolemia |  |  |  |  |  |
| case, n | 105 | 262 | 61 | 453 |  |
| Multivariable-adjusted Model 1 | ref (1.000) | 0.924 (0.727–1.174) | 1.116 (0.798–1.560) | 0.948 (0.726–1.237) | 0.6517 |
| Multivariable-adjusted Model 2 | ref (1.000) | 0.919 (0.722–1.170) | 1.097 (0.784–1.534) | 0.944 (0.722–1.233) | 0.8205 |
| Multivariable-adjusted Model 3 | ref (1.000) | 0.925 (0.727–1.176) | 1.110 (0.794–1.553) | 0.955 (0.731–1.247) | 0.5543 |
| Dyslipidemia |  |  |  |  |  |
| case, n | 139 | 349 | 84 | 605 |  |
| Multivariable-adjusted Model 1 | ref (1.000) | 0.925 (0.751–1.138) | 1.117 (0.838–1.489) | 0.916 (0.727–1.154) | 0.4693 |
| Multivariable-adjusted Model 2 | ref (1.000) | 0.924 (0.750–1.139) | 1.103 (0.827–1.471) | 0.916 (0.727–1.156) | 0.5943 |
| Multivariable-adjusted Model 3 | ref (1.000) | 0.929 (0.754–1.144) | 1.115 (0.836–1.486) | 0.926 (0.734–1.167) | 0.3768 |
|  |  |  |  |  |  |
| Women (n = 2,736) | 401 | 951 | 225 | 1,159 |  |
| Median (range), serving/day | 0.4 (0.00-0.79) | 1.5 (1.00–1.50) | 2 (2.00–2.00) | 3 (3.00–4.50) |  |
| Person year, mean (sum) | 8.2 (2542.3) | 8.5 (6401.7) | 7.8 (1355.9) | 8.4 (7584.2) |  |
| Hypertriglyceridemia |  |  |  |  |  |
| case, n | 83 | 192 | 54 | 259 |  |
| Multivariable-adjusted Model 1 | ref (1.000) | 0.806 (0.613–1.059) | 1.111 (0.774–1.596) | 0.870 (0.641–1.182) | 0.3052 |
| Multivariable-adjusted Model 2 | ref (1.000) | 0.807 (0.613–1.061) | 1.104 (0.769–1.587) | 0.872 (0.769–1.185) | 0.3046 |
| Multivariable-adjusted Model 3 | ref (1.000) | 0.809 (0.615–1.065) | 1.118 (0.778–1.607) | 0.876 (0.645–1.190) | 0.5892 |
| Hyper-LDL-cholesterolemia |  |  |  |  |  |
| case, n | 118 | 289 | 84 | 356 |  |
| Multivariable-adjusted Model 1 | ref (1.000) | 0.961 (0.764–1.208) | 1.226 (0.909–1.655) | 1.011 (0.775–1.319) | 0.7869 |
| Multivariable-adjusted Model 2 | ref (1.000) | 0.963 (0.765–1.211) | 1.241 (0.919–1.675) | 1.016 (0.778–1.325) | 0.6696 |
| Multivariable-adjusted Model 3 | ref (1.000) | 0.962 (0.765–1.210) | 1.232 (0.912–1.664) | 1.013 (0.776–1.322) | 0.4847 |
| Hypercholesterolemia |  |  |  |  |  |
| case, n | 110 | 263 | 81 | 331 |  |
| Multivariable-adjusted Model 1 | ref (1.000) | 0.937 (0.749–1.171) | 1.383 (1.037–1.843) | 1.005 (0.810–1.247) | 0.7016 |
| Multivariable-adjusted Model 2 | ref (1.000) | 0.992 (0.780–1.260) | 1.362 (0.999–1.857) | 1.131 (0.855–1.496) | 0.5381 |
| Multivariable-adjusted Model 3 | ref (1.000) | 0.990 (0.779–1.259) | 1.346 (0.987–1.836) | 1.126 (0.851–1.490) | 0.1314 |
| Hypo-HDL-cholesterolemia |  |  |  |  |  |
| case, n | 146 | 352 | 84 | 432 |  |
| Multivariable-adjusted Model 1 | ref (1.000) | 0.781 (0.651–0.981) | 0.955 (0.719–1.267) | 0.781 (0.620–0.985) | 0.6523 |
| Multivariable-adjusted Model 2 | ref (1.000) | 0.805 (0.656–0.988) | 0.969 (0.730–1.285) | 0.792 (0.628–0.998) | 0.7890 |
| Multivariable-adjusted Model 3 | ref (1.000) | 0.806 (0.657–0.990) | 0.975 (0.734–1.294) | 0.794 (0.630–1.001) | 0.0969 |
| Dyslipidemia |  |  |  |  |  |
| case, n | 242 | 576 | 142 | 722 |  |
| Multivariable-adjusted Model 1 | ref (1.000) | 0.858 (0.731–1.007) | 1.004 (0.806–1.251) | 0.889 (0.740–1.068) | 0.4699 |
| Multivariable-adjusted Model 2 | ref (1.000) | 0.859 (0.732–1.009) | 1.015 (0.815–1.264) | 0.894 (0.744–1.074) | 0.5009 |
| Multivariable-adjusted Model 3 | ref (1.000) | 0.859 (0.732–1.009) | 1.015 (0.815–1.265) | 0.894 (0.744–1.074) | 0.6412 |

One serving size of baechu kimchi was defined as 50 g.

Multivariable-adjusted Model 1 was adjusted for age, BMI, income level, education level, marital status, smoking status, alcohol consumption, physical activity, energy intake, rice intake, energy-adjusted sodium intake, and fruit intake.

Multivariable-adjusted Model 2 was adjusted for age, BMI, income level, education level, marital status, smoking status, alcohol consumption, physical activity, energy intake, rice intake, energy-adjusted sodium intake, medications (hypertension, insulin), and dietary supplements.

Multivariable-adjusted Model 3 was adjusted for age, BMI, income level, education level, marital status, smoking status, alcohol consumption, physical activity, energy intake, rice intake, energy-adjusted sodium intake, fruit intake, medications (hypertension, insulin), and dietary supplements.

The p for trend was calculated by assigning median values to each group and was treated as a continuous variable.

^1^LDL, low-density lipoprotein.

^2^HDL, high-density lipoprotein.

**Supplementary Table 3.** Stratified analysis of the association between baechu kimchi consumption and dylipidemia by BMI and age.

|  | Baechu kimchi consumption (g/day) | | |
| --- | --- | --- | --- |
|  | <1 serving/day | ≥3 servings/day | P for interaction |
| Men (n = 1,930) |  |  |  |
| Hypertriglyceridemia |  |  |  |
| BMI^1^ |  |  |  |
| < 25 | ref (1.000) | 0.905(0.587-1.395) | 0.5358 |
| ≥ 25 | ref (1.000) | 0.890 (0.461-1.717) |  |
| Age (Median) |  |  |  |
| < 50 | ref (1.000) | 0.671 (0.409-1.103) | 0.5013 |
| ≥ 50 | ref (1.000) | 0.956 (0.569-1.605) |  |
| Hyper-LDL^2^-cholesterolemia |  |  |  |
| BMI |  |  |  |
| < 25 | ref (1.000) | 0.765 (0.454-1.288) | 0.9997 |
| ≥ 25 | ref (1.000) | 0.808 (0.351-1.859) |  |
| Age (Median) |  |  |  |
| < 50 | ref (1.000) | 0.851 (0.429-1.688) | 0.7570 |
| ≥ 50 | ref (1.000) | 0.684 (0.383-1.222) |  |
| Hypercholesterolemia |  |  |  |
| BMI |  |  |  |
| < 25 | ref (1.000) | 0.762 (0.424-1.370) | 0.5308 |
| ≥ 25 | ref (1.000) | 0.595 (0.245-1.445) |  |
| Age (Median) |  |  |  |
| < 50 | ref (1.000) | 0.654 (0.324-1.320) | 0.4997 |
| ≥ 50 | ref (1.000) | 0.566 (0.284-1.128) |  |
| Hypo-HDL^3^-cholesterolemia |  |  |  |
| BMI |  |  |  |
| < 25 | ref (1.000) | 0.936 (0.865-1.285) | 0.1270 |
| ≥ 25 | ref (1.000) | 0.975 (0.582-1.634) |  |
| Age (Median) |  |  |  |
| < 50 | ref (1.000) | 0.648 (0.429-0.979) | 0.6606 |
| ≥ 50 | ref (1.000) | 1.115 (0.786-1.581) |  |
| Dyslipidemia |  |  |  |
| BMI |  |  |  |
| < 25 | ref (1.000) | 0.938 (0.712-1.235) | 0.5432 |
| ≥ 25 | ref (1.000) | 0.866 (0.556-1.351) |  |
| Age (Median) |  |  |  |
| < 50 | ref (1.000) | 0.692 (0.485-0.989) | 0.5171 |
| ≥ 50 | ref (1.000) | 1.002 (0.738-1.359) |  |
|  |  |  |  |
| Women (n = 2,736) |  |  |  |
| Hypertriglyceridemia |  |  |  |
| BMI |  |  |  |
| < 25 | ref (1.000) | 1.053 (0.686-1.617) | 0.0300 |
| ≥ 25 | ref (1.000) | 0.647 (0.413-1.016) |  |
| Age (Median) |  |  |  |
| < 49 | ref (1.000) | 0.730 (0.462-1.154) | 0.0681 |
| ≥ 49 | ref (1.000) | 1.038 (0.676-1.595) |  |
| Hyper-LDL-cholesterolemia |  |  |  |
| BMI |  |  |  |
| < 25 | ref (1.000) | 0.934 (0.668-1.308) | 0.6069 |
| ≥ 25 | ref (1.000) | 1.077 (0.689-1.681) |  |
| Age (Median) |  |  |  |
| < 49 | ref (1.000) | 1.019 (0.702-1.478) | 0.1808 |
| ≥ 49 | ref (1.000) | 0.980 (0.665-1.444) |  |
| Hypercholesterolemia |  |  |  |
| BMI |  |  |  |
| < 25 | ref (1.000) | 1.119 (0.790-1.585) | 0.8967 |
| ≥ 25 | ref (1.000) | 1.055 (0.651-1.707) |  |
| Age (Median) |  |  |  |
| < 49 | ref (1.000) | 1.285 (0.864-1.911) | 0.2272 |
| ≥ 49 | ref (1.000) | 0.981 (0.654-1.471) |  |
| Hypo-HDL-cholesterolemia |  |  |  |
| BMI |  |  |  |
| < 25 | ref (1.000) | 0.703 (0.511-0.966) | 0.8371 |
| ≥ 25 | ref (1.000) | 0.857 (0.601-1.223) |  |
| Age (Median) |  |  |  |
| < 49 | ref (1.000) | 0.661 (0.469-0.932) | 0.6596 |
| ≥ 49 | ref (1.000) | 0.899 (0.650-1.242) |  |
| Dyslipidemia |  |  |  |
| BMI |  |  |  |
| < 25 | ref (1.000) | 0.864 (0.679-1.099) | 0.7716 |
| ≥ 25 | ref (1.000) | 0.875 (0.652-1.175) |  |
| Age (Median) |  |  |  |
| < 49 | ref (1.000) | 0.831 (0.638-1.082) | 0.6503 |
| ≥ 49 | ref (1.000) | 0.933 (0.719-1.212) |  |

One serving size of baechu kimchi was defined as 50 g.

Model was adjusted for age, age median groups (men: < 50 or ≥ 50 years and women: < 49 or ≥ 49 years), BMI, BMI groups (< 25 or ≥ 25 kg/m^2^), income level, education level, marital status, smoking status, alcohol consumption, physical activity, energy intake, rice intake, energy-adjusted sodium intake.

^1^BMI, body mass index.

^2^LDL, low-density lipoprotein.

^3^HDL, high-density lipoprotein.
